# Innovative 3D Model of the Human Middle Ear in High Resolution with a Histological Microgrinding Method: A Feasibility Study and Comparison with μCT

**DOI:** 10.1155/2017/6753604

**Published:** 2017-05-03

**Authors:** Susanne Bradel, Laura Doniga-Crivat, Silke Besdo, Franziska Lexow, Michael Fehr, Thomas Lenarz, Nils Prenzler, Gudrun Brandes

**Affiliations:** ^1^Department of Otolaryngology, Hannover Medical School, Hannover, Germany; ^2^Clinic for Exotic Pets, Reptiles, Pet, and Feral Birds, University of Veterinary Medicine Hannover, Hannover, Germany; ^3^Institute of Continuum Mechanics, Gottfried Wilhelm Leibniz Universität, Hannover, Germany; ^4^Institute for Neuroanatomy and Cell Biology, Hannover Medical School, Hannover, Germany

## Abstract

*Conclusion*. The development of a histological 3D model of the tympanic cavity visualizes the exact microanatomy of the sound conduction organ and is therefore essential for finite elements simulations and surgical training.* Objectives*. So far, no accurate histological 3D model of the sound conduction system existed in literature. For 3D reconstruction of the very fine structures inside and outside the auditory ossicles, a method based on histological slices allows a more differential analysis of both hard and soft tissues and could thus be superior to *μ*CT.* Method*. A complete temporal bone was embedded in epoxy resin and microground in distances of about 34 *μ*m. After photodocumentation of every plane, a 3D reconstruction was performed by using the Computer Aided Design (CAD) program Rhinoceros 5®. For comparison, a *μ*CT of the same specimen resulted in a 3D model of the calcified structures in the middle ear.* Results*. The histological 3D model gives an excellent overview to all anatomical soft and bony tissues of the human auditory ossicles. Specifically the fine blood vessel system and the exact dimension of cartilage areas inside the ossicles can be illustrated much more precisely than with *μ*CT data. The present technique also allows the evaluation of the fine connecting ligaments inside the tympanic cavity.

## 1. Introduction

The middle ear can be affected by a number of pathologies which may lead to hearing loss. To generate physiologically optimized middle ear prostheses, as well as a support for advanced middle ear surgery, the knowledge of the microanatomy of the middle ear is essential. In understanding the physiological conditions of the blood supply inside the tympanic cavity, complications in the middle ear surgery, for example, the necrosis of the long process of the* incus* after* stapes* prosthesis surgery, can be explained.

In the middle of the 16th century, the anatomists Andreas Vesalius (1514–1564) and Giovanni Filippo Ingrassia (1510–1580) discovered the auditory ossicles in the tympanic cavity of the human petrous portion. Ingrassia identified the* stapes* and Vesalius the* incus* and* malleus* [1, 2]. These ossicles are interconnected through a saddle shaped* diarthrosis* between* malleus* und* incus* as well as an* enarthrosis* between* incus* and* stapes* [3]. The* malleus* is fixed with its manubrium to the ear drum and the* stapes* with its footplate to the oval window. Therefore they bridge the gap between the external and internal ear, acting as sound conduction organ.

In the 20th century several scientists examined the three-dimensional (3D) structure of the human auditory ossicles with the following methods.

Oesterle [4] decalcified thirty temporal bones from embryos up to 66-year-old humans and cut them in 15–25 *μ*m thick slices, but not every section was analyzed. After staining with hematoxylin and eosin the bone composition was evaluated. The distribution of the collagen fibers was examined with a polarization microscope.

G. T. Nager and M. Nager [5] included temporal bones from 110 humans, which were aged between 20 and 43 years in their study. They cut the specimens horizontally with a thickness of 24 *μ*m per slice and analyzed them in an interval of 120 *μ*m or 240 *μ*m to evaluate the blood supply of the ossicles in the tympanic cavity.

Hamberger et al. [6] examined the blood supply and the blood vessels in the mucosa of the auditory ossicles with another technique: complete temporal bones as well as extracted auditory ossicles were fixed with formalin and then exposed to a solution containing benzidine, alcohol, and hydrogen peroxide, which stained the blood cells and then they analyzed the specimens with binocular glasses.

Anson and Winch [7] examined two human temporal bones, one from a newborn and the other from a 19-year-old man to evaluate the contents of the auditory ossicles. The decalcified bone was cut into transversal sections and photodocumented with a magnification factor of 22. A 3D reconstruction of the channel system inside the ossicles was performed on card board with an enlargement factor of 60.

In the attempt to perform a highly accurate simulation of the sound conduction system, a review of the literature revealed that there is no anatomical correct 3D model according to the state of technology. The results mentioned above, obtained by using cutting sections of decalcified tissue or separated auditory ossicles, are rather imprecise compared with today's technological possibilities. New models are necessary for optimizing middle ear prosthesis [8]. The wide interval between the examined slices is problematic for the reconstruction of the structures in between [9]. Understanding the physiological conditions of the blood supply in the auditory ossicles and the tympanic cavity is the crucial first step in preventing complications in middle ear surgery.

The presented study developed a new, innovative, and more detailed microgrinding method of the human middle ear to produce a computer supported 3D model of the in situ positioned, anatomically correct ossicular chain in the tympanic cavity. The advantage of the technique is the high resolution of the model based on the very short intervals between the examined surfaces. But the time consuming pilot study had to be restricted to only one specimen in order to prove its superiority to the available 3D reconstructions in the literature. The attempt of the presented study is not to provide general statements on the microanatomy of the human middle ear but to introduce a very exact 3D model in high resolution based on histological sections and manual segmentation in terms of a proof of feasibility study. The analysis of the histology of the grinded planes revealed detailed information of the tissues involved, which were not apparent in the micro CT. As CT scans are probably the fastest way to gather data for 3D models or for finite element simulations, the informative value of this method has to be compared with CT based models. If the superiority of the 3D model based on the microgrinding can be demonstrated, an increase of man and machine power for detailed analysis of more samples of the human middle ear will be possible in the future.

## 2. Materials and Methods

### 2.1. Sample Preparation

A left human temporal bone was taken from a body donor (56-year-old man), who gave face-to-face and in free will his informed written consent (own testament during his lifetime) to bequeath his body for teaching and research at the university after his death within the body donation program of the Institute of Functional and Applied Anatomy, Hannover Medical School, Hannover, Germany. The sample was lesion-free and without pathologies. It was fixed in a mixture of 2.5% glutardialdehyde in 0.1 M sodium cacodylate buffer with pH 7.3 (Merck, Darmstadt, Germany) at 4°C for three days. Afterwards a dehydrating process was performed using a four-step ethanol series with increasing concentration, and the sample was then dried over night at 65°C in a drying cabinet. Subsequently, the fixed and dehydrated sample was embedded in uncolored epoxy resin (SpeciFix 20 Kit, Struers A/S, Rodoyre, Denmark) under vacuum conditions. To also fill the tympanic cavity with epoxy resin, a hole was pierced into the tympanic membrane with a needle. After drying, three holes were drilled vertically into the epoxy resin and filled with wooden sticks to serve as reference marks. To ensure the holes were drilled exactly vertically into the epoxy resin, this process was conducted at a lathe.

Additionally, one series of separated right ear auditory ossicles from the anatomic collection of the Department of Otolaryngology, Hannover Medical School, Germany, was used for *μ*CT examination. These ossicles were extracted from another adult donor without ear pathologies and were fixed in formalin solution.

### 2.2. *μ*CT Imaging

The epoxy resin embedded left human temporal bone as well the formalin-fixed separated ossicular chain of a right ear was examined with a *μ*CT (Scanco Medical *μ*CT 80, Desktop Cone-Beam Micro-CT Scanner) after being embedded in epoxy resin. The datasets of both specimens were exported into DICOM (.dcm) format.

### 2.3. Microgrinding Imaging

For the microgrinding, the embedded left human temporal bone was ground and polished with a grinding machine (Buehler beta with vector, with Lc Power Head, Buehler GMBH, Germany) and grinding paper P2500 (medium grain size 10 *μ*m, Buehler GMBH) as well as P4000 (medium grain size 5 *μ*m, Buehler GMBH). The grinding direction was in frontal plane, horizontal to the tympanic membrane, and started at the bottom of the* mastoid antrum*. Thus the first structure which was cut horizontally is the tip of the short* crus* of the* incus* and the* pars flaccida* of the tympanic membrane.

In every grinding run the abrasion of the sample was measured in three places near the reference marks by the thickness of the remaining sample with a digital micrometer caliper. Consequently, the abrasion of the sample in every grinding run was about 35 *μ*m in the area of the ossicles and about 94 *μ*m in the periphery atop and beneath the ossicles.

The surface of the sample was stained for two minutes with the modified method according to Mann-Dominici [10]: after incubation with an acid cytoplasmic stain of 0.1% eosin (Certistain, Merck) and 0.25% Orange G (Certistain, Merck) in 50% ethanol for 2 min and rinsing with Aqua dest, an alkaline nuclear stain by 0.5% Toluidine blue (Sigma-Aldrich Corp., St. Louis, Montana, USA) in 50% ethanol was performed for 2 min and stopped by rinsing with Aqua dest.

A total of 151 layers were ground and stained. The histological images were taken with a digital camera system (AxioCam MRc, Zeiss, Jena, Germany), attached to a microscope (Zeiss, SteREO Discovery.V20) in 4- to 100-fold magnification, and illuminated from an external cold light source. In total 20996 histological images were taken documenting the histological structures in different magnifications. With Adobe Photoshop (Adobe software Ireland Ltd.) every histological high magnification microscopic image of the fine structures was placed into the histological overview image of the same layer. Thus an image of the hard and soft tissues in high resolution together with orientation marks was obtained of every layer and saved as JPEG (.JPG).

### 2.4. 3D Modelling

For segmentation the data sets of the *μ*CT examinations of the embedded sample the loose ossicles were loaded one after another into the biomedical software Mimics® (Materialise HQ, Belgium). With the software 3-matrics® (Materialise HQ, Belgium) the images were adapted and saved in Standard Tessellation Language (.STL) and transformed into Nonuniform Rational B-Splines (NURBS) with the software Geomagic Studio 2012 (Geomagic, Inc., USA) and saved as Initial Graphics Exchange Specification format (.IGES). With this method the 3D reconstructions of both specimens were realized.

**Figure 1:**
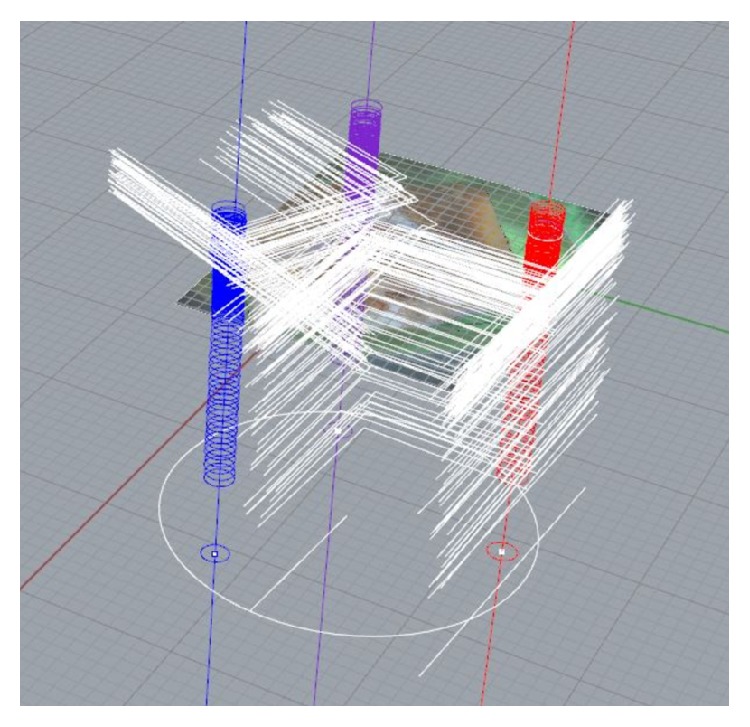
Working surface of the CAD program Rhinoceros 5. The markings of the reference marks are arranged in piles and colored in red, blue, and lilac. The adjustment lines for the microgrinding images are marked in white.

The 3D model of the auditory ossicles created from the histological images was designed with the CAD program Rhinoceros 5 (64-bit; McNeel). Therefore the reference marks were replaced by rings at the CAD program surface (Figure 1). These rings were arranged in piles in the interval of the measured abrasion that occurred during the grinding runs at these points. Every histological image could then be loaded into the program and adjusted through the reference marks to ensure its right direction in the image pile. Due to minor skew positions of the grinding machine, the images sometimes had to be loaded in inclined positions into the program to guarantee the right adjustment. With these adjustment methods nearly every error index of the microgrinding could be eliminated for accurate designing of a 3D model.

The generated model consists of the outer shape of the ossicles with its ligaments, muscles, the articular capsules of the ossicular joints, and the articulation cartilage on the joint surface. The inner structures of the ossicles, that is, the inner vascular system and cartilage areas, were also modeled. Therefore every histological image was loaded into the program and adjusted accordingly to the reference marks and then structures of interest were marked with lines. Finally, these lines were connected to each other and a freeform surface was generated.

The *μ*CT models and the 3D model from the histological images were meshed with tetrahedrons. The volumes of the tetrahedrons were added together to get the volumes of the different structures and the total volume of the ossicles.

## 3. Results and Discussion

In this study an embedded specimen of a left adult human temporal bone was examined by the microgrinding method with removal of circa 35 *μ*m per grinding run in the region of the auditory ossicles. This fine working and the high resolution of the 20996 histological images taken in total during the microgrinding process allow not only a very detailed histological examination of the images, but also an anatomically correct and highly accurate 3D reconstruction of the structures inside the tympanic cavity. Based on the pilot study character and the time consuming steps of procedure of this project, the number of samples was restricted to one. Unfortunately, the analyzed planes are lost during the grinding process to reveal the deeper layer of the tissue block. However, this innovative new preparation method produces a 3D model of the microanatomic structures in a very high resolution, whereas the model examined in a *μ*CT can only differentiate between calcified and not calcified structures.

### 3.1. Histological Examination

The histological images of the microgrinding surface as well the deeper transparent zones of the embedded middle ear show that not only the tympanic cavity but also the ossicular chains are covered by a thin layer of mucosa. These mucosal membranes are connected to each other by several thin plications, which contain the ligaments as well as blood vessels. The blood vessel system forms a fine network within the mucosal layer around the ossicles.

#### 3.1.1. Blood Supply around Auditory Ossicles

**Figure 2:**
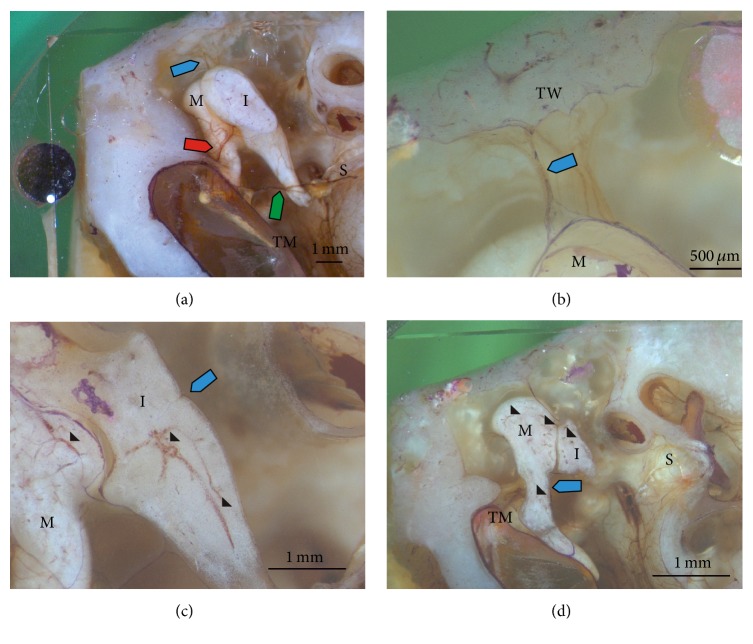
Reflected light microscopic images of the grinding surface after surface staining with modified staining according to Mann-Dominici. (a) Inside the tympanic cavity the stained tympanic membrane (TM) and* incus* (I), as well the deeper lying* malleus* (M) and* stapes* (S), can be seen. The blood supply is guaranteed by a branch of the anterior tympanic artery inside the plication of the superior malleolar ligament (blue arrow) and inside the anterior malleolar plication with its dividing point to the* malleus* and* incus* (red arrow). Additionally, the posterior tympanic artery runs over the incudostapedial joint and emits a branch to the* malleus* (green arrow). In (b) the superior branch of the anterior tympanic artery follows the plication of the superior tympanic ligament from the tympanic wall (TW) to the head of the* malleus* (blue arrow). In (c) the entrance of a branch of the posterior tympanic artery emitting to the* incus* on its medial side through a nutrient* foramen* is depicted (blue arrow) as well as the blood vessel system inside the* malleus* and* incus* (arrowhead). Only the cartilage area inside the* incus* (asterisk) contains no vessels. In (d) the entrance of a branch of the posterior tympanic artery through a nutrient* foramen* on the medial side of the* malleus* can be seen (blue arrow) as well as the blood vessel system inside the malleus and incus (arrowhead). Grinding surfaces after removing (a) 6.106 mm, (b) 7.460 mm, (c) 7.360 mm, and (d) 8.001 mm of the total sample, respectively.

The major blood supply of the* malleus* and* incus* can be followed constantly through every plane, because the deeper course can also be tracked inside the embedded specimen, due to the uncolored epoxy resin. It is the anterior tympanic artery, which is a branch of the maxillary artery and enters the middle ear through the petrotympanic fissure. This artery divides into two main nutrient branches: one branch runs inside the anterior malleolar plication and at this point also divides into two vessels: one for the* malleus* and the other for the blood supply of the* incus* (Figure 2(a)). Before the main nutrition branch enters the anterior malleolar plication, it also emits a vascular circle around the tympanic membrane. The other branch of the anterior tympanic artery runs to the superior malleolar ligament and reaches the* malleus* through the plication of this ligament at the head of the* malleus* (Figure 2(b)).

The malleolar branch of the artery inside the anterior malleolar plication runs along the anterior process of the* malleus* to the lateral part of the* malleus*' neck and penetrates the bone through two nutrition* foramina*, connecting with the fine vessel system inside the ossicle (Figure 2(a)). In addition, the posterior tympanic artery also branches to the* malleus*. This branch leaves the posterior tympanic artery near the section where the chorda tympani crosses the tympanic cavity along the medial part of the* malleus*' neck. It runs only shortly along the bone and then penetrates it through a nutrition* foramen* on the medial side of the* malleus* (Figure 2(d)).

Three blood vessels penetrate the* incus* through nutrition* foramina*. The main blood vessel derives from the incudal branch of the anterior tympanic artery, which runs through the anterior malleolar plication (Figure 2(a)). This blood vessel reaches the* incus* on the lateral side of the body, near the base of the long* crus* and close to the articulation area. From the posterior tympanic artery, a branch with large diameter leaves the blood vessel near the incudostapedial joint (Figure 2(a)) and runs on the medial side of the* incus*' mucosa to the base of the long* crus* where it enters the bone through a nutrition* foramen* (Figure 2(c)). The third main blood vessel reaches the* incus* on the medial side at the base of the short* crus* and penetrates the bone through another nutrition* foramen*. This blood vessel is a branch of the superior main branch of the anterior tympanic artery and runs through the plication of the superior incudal ligament.

The main blood supply of this specimen concurs with the findings of G. T. Nager and M. Nager [5], Hamberger [6, 11], and Anson and Winch [7]. These main blood vessels branch out into an extensive network of mucosal nutrient vessels on the surface of the auditory ossicles. For Hamberger et al. [6] this mucosal network is particularly important for the vascularization of the processes of the* incus* and* malleus*, but in their study the blood vessel system inside the ossicles was not examined. Additionally, they used loosened ossicles in their study to examine the mucosal blood vessel system of every single ossicle. For the examination of the main blood supply they studied the specimen before extracting the ossicles from the tympanic cavity, which limits the perspective and causes an undetectable back side of the ossicular chain. In the here presented study the blood supply of the auditory ossicles can be followed from its entrance in the temporal cavity to the mucosal layer around the auditory ossicles or inside the auditory ossicles.

The blood supply of the* stapes* varies from the other two ossicles. The posterior tympanic artery runs over the incudostapedial joint and gives a rich blood vessel system to the mucosa of the stapedial head. From this network one bigger blood vessel and some of its smaller branches run along the inner side of the u-shaped anterior and posterior* crus* in direction of the footplate of the* stapes*. In the surrounding tissue of the stapedial muscle tendon runs two blood vessels which reach the* stapes* at the connection point of the tendon and then continue in the mucosal layer of the* stapes* into the direction of the stapedial head and to the anterior* crus*. The branch leading to the head of the* stapes* penetrates the bone through a nutrition* foramen* on the anterior side of the head and runs through the bone, leaving it to join the blood vessel on the inner side of the u-shaped anterior and posterior* crus* of the* stapes*. On the footplate of the* stapes* there are only a few fine blood vessels within the mucosal layer. Along the annular ligament of the* stapes* a vascular ring is located.

The blood supply of the* stapes* in previous studies also concurs with the findings in our study: G. T. Nager and M. Nager [5] explained this with the thickness of the* stapes crura*. The thickness of the u-shaped* crura*, measured from the outer to the inner face, is similar to the thickness of Haversian lamellar system in other bones. Therefore the* crura* are nourished through diffusion from the network of mucosal blood vessels. In lamellar bone the maximal diffusion distance is about 150 *μ*m [11]. With the 3D model of our histological images it is possible to measure the thickness of the ossicular chain. The thickness of the stapedial* crura* from the outer to the inner surface is between 103 *μ*m and 134 *μ*m, which means that it is much shorter than the maximal diffusion distance in bone. This explains why the* stapes* has no blood vessel system inside the bone as it is nourished through the mucosal blood vessel network.

#### 3.1.2. Inner Vascular Structures of the Auditory Ossicles

**Figure 3:**
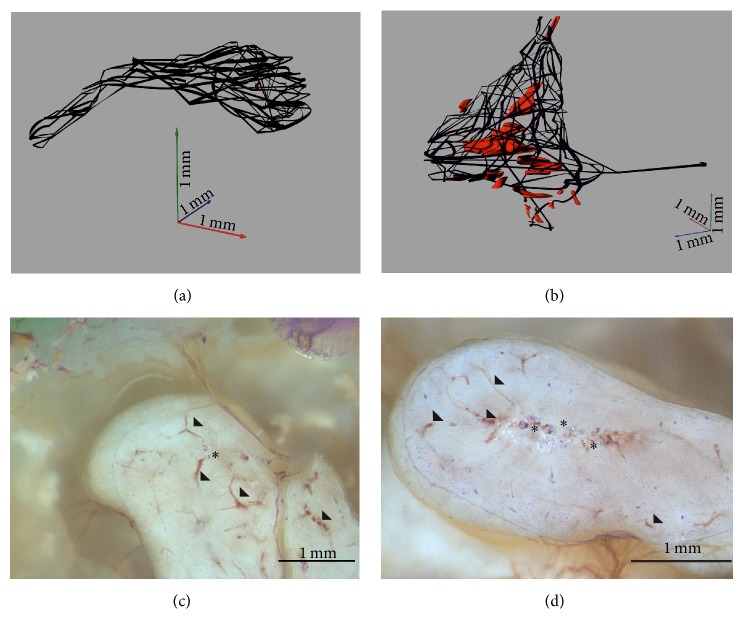
The blood vessel system inside the* malleus* (a, c) and* incus* (b, d) of the 3D model of the stained histological planes (a, b) compared with the selected planes (c, d). The blood vessel system (black) and the small cartilage areas (red) inside the* malleus* (a) and* incus* (b) are reconstructed three-dimensionally. Exemplary reflected light microscopic images of the grinding surface after surface staining with the modified staining according to Mann-Dominici demonstrate the blood vessels (arrowhead) and cartilage (asterisk) inside the* malleus* head (c) and* incus* body (d). Grinding surfaces after removing (c) 7.893 mm and (d) 6.565 mm of the total sample, respectively.

With the novel microgrinding technique blood vessels can be found not only in the mucosa around the ossicular chain but also inside the ossicles. Some nutrient vessels penetrate the bones of* malleus* and* incus* on defined regions to build a fine vascular network inside these ossicles. These blood vessels form a second blood vessel system inside the auditory ossicles. A higher density of blood vessels can be found in the head and neck compared with the other parts of the* malleus* (Figures 3(a) and 3(c)). The amount of blood vessels inside the* incus* is higher than in the* malleus*. In the long* crus* of the* incus* only two blood vessels can be found, which reach into the lenticular process (Figures 3(b) and 3(d)). The* stapes* is penetrated only by one blood vessel, which reaches the anterior side of the head by following the tendon of the stapedial muscle. On the posterior side this blood vessel splits into the mucosal blood vessel system.

Chen et al. [12] and Zenev et al. [13] as well as Hassmann and Chodynicki [14] examined extracted single auditory ossicles by scanning electron microscopy. The penetrating nutrient holes they found in the surface of the auditory ossicles can clearly be seen in our 3D model of the *μ*CT of the embedded specimen of our study as well as of the single scanned not embedded ossicles. The inner vascular network of the ossicles can also be supported by scanning microscopy of randomly fractured planes of the ossicles [6, 11].

Nevertheless, by using uncolored epoxy resin and ground resin and grinding in very thin layers we can follow the course of the internal vessels of the ossicles through the nutrition pores into the vascular network inside the “mesenteries” of the tympanic cavity as well as in the mucosa covering the auditory ossicles.

#### 3.1.3. Cartilage Areas inside the Bone

By examining the grinding sections only one cartilaginous area can be found within the head of the* malleus* (Figures 3(a) and 3(d)). The occurrence of cartilage inside the* incus*, however, is much higher. Most of these cartilage areas are located in the* incus* body near to the short* crus* and the articulation area of the incudomalleolar joint (Figures 3(b) and 3(d)). Several other cartilage areas can also be found inside the short* crus*. There is no sign of cartilage in the long* crus* of the* incus* as well as inside the* stapes*.

Oesterle [4] also found cartilage areas inside auditory ossicles and explained their existence by the remaining of cartilage deposits during the ossification process. He described that these “interglobular spaces” are always present but variable in their amount. They are located in several preferential areas in the handle and the anterior process of the* malleus*, the short process of the* incus*, and also sometimes at the border of the* stapes*' footplate. Oesterle's findings inside the* incus* are therefore comparable to the specimen of our study.

Anson [5] discovered a persistent marrow cavity inside the* incus.* However, by analyzing the histological images of every section of the auditory ossicles in our specimen, we did not find any marrow spaces.

The appearance of marrow spaces and cartilage areas can be explained by the embryological development of the auditory ossicles. From the mesenchyme of the first branchial arch the head and neck of the* malleus* as well as the body of the* incus* descend. Converting the cartilage to the endosteal bone, primitive bone marrow spaces arise. By the 22nd week of gravidity, the ossification in* malleus* and* incus* expands to all parts of the ossicles, except for the incudomalleolar joint region, the short process of the* incus*, and the handle of the* malleus* [15]. By that stage, the head of the* malleus* and the body of the* incus* still contain primitive marrow spaces whereas those in the* malleus* and* incus* disappear completely before the age of 25 months [16].

### 3.2. 3D Reconstructions

#### 3.2.1. 3D Model of the *μ*CT Data of Single Scanned Ossicles

**Figure 4:**
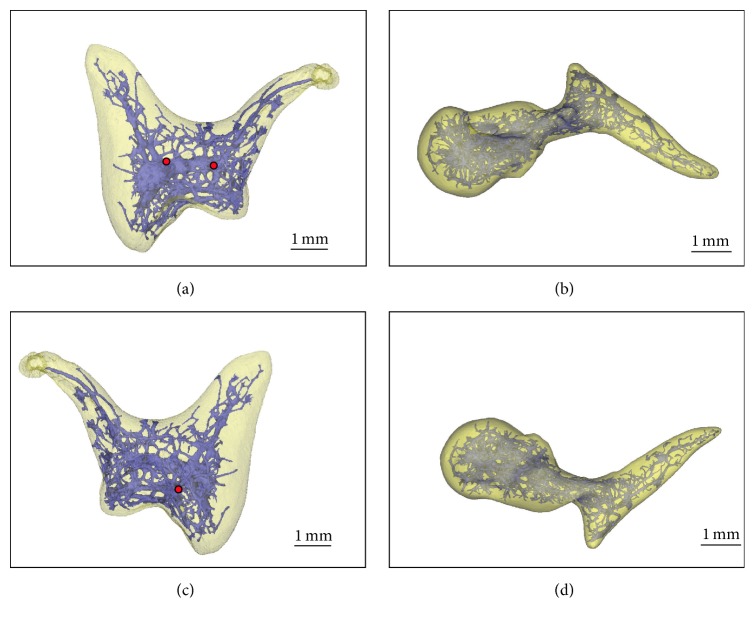
3D models of the *μ*CT data of the separated scanned* incus* (a, c) and* malleus* (b, d) in the lateral (a, b) and medial aspect (c, d), respectively. In the half-transparent ossicles the bone structure is colored in yellow, the not calcified inner structures are colored in blue, and nutrient* foramina* are marked in red.

To expound possible supremacy of the time consuming grinding method, the information value was compared with a relatively easy and fast *μ*CT analysis of the conserved specimen. But the generated 3D model of the *μ*CT examination of the separated auditory ossicles can only depict the calcified areas of the bones. The not calcified segments inside the ossicles only fused to an arborescent system with a central core region in the body of the* incus, whereas the* ramified system in the* malleus* reveals a denser part in the head and neck (Figure 4). In the manubrium of the* malleus* fine channels run in direction of the* umbo* branching out into the periphery and ending close to the surface of the bone. In the short* crus* of the* incus* the channel in direction of the bone's surface resembles a bottle brush, whereas in the long* crus* of the* incus* only one pipe runs into the direction of the articulation process (Figures 4(a) and 4(c)). Only three tubes reach the surface of the* incus*: one on the lateral side of the body near the base of the long* crus* and close to the articulation area, one on the short* crus* (Figure 4(a)), and another one on the medial side at the base of the short* crus* (Figure 4(c)).

#### 3.2.2. 3D Model of the *μ*CT Data of the Embedded Temporal Bone

**Figure 5:**
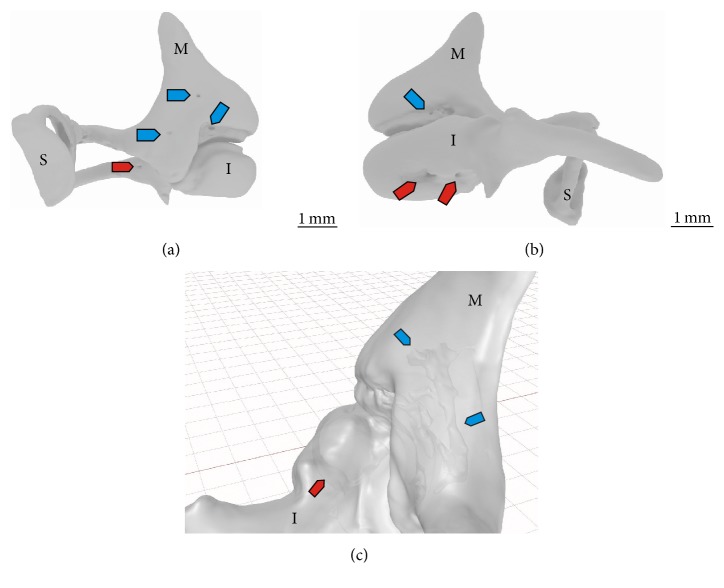
3D model of the surface of the* malleus* (M),* incus* (I), and* stapes* (S) reconstructed by the *μ*CT dataset of the embedded specimen in the following orientations: medial (a) and lateral surface (b) of the auditory ossicles with the nutrient* foramen* on the* malleus* (blue arrow) and on the* incus* (red arrows). From the lateral side (c) the not calcified areas can be seen inside the half-transparent* malleus* (blue arrow) and* incus* (red arrows).

The generated 3D model of the embedded specimen illustrates the ossicular chain in their anatomically correct orientation within the temporal cavity as well as their relation to each other. The nutrient openings in the bony surface of the ossicles can here clearly be seen. One* foramen* is located on the medial side of the* malleus* neck (Figure 5(a)). Two nutrient* foramina* can be found on the lateral side of the* malleus'* neck (Figure 5(b)). The* incus* shows three nutrient* foramina*: one on the lateral side of the* incus* body near the articulation area with the* malleus* (Figure 5(b)), one on the medial side of the base of the long* crus*, and the third one also on the medial side of the* incus* but on the base of the short* crus* (Figure 5(a)). On some, but not all nutrient openings, the connection with the inner not calcified tubules is obvious (Figure 5(c)).

The volume of the whole ossicles and especially of the not calcified structures can be evaluated. The total volume of the* malleus* is 11800 *μ*m^3^ and 0.35% of the tissue is not calcified. The* incus* is not calcified in 1.82% of the total volume of 14240 *μ*m^3^. The totally calcified* stapes* has a volume of 1660 *μ*m^3^.

#### 3.2.3. 3D Model of the Histological Analysis of the Embedded Temporal Bone

In comparison, the histological data set allows a differentiated analysis of all cellular and extracellular structures of the ossicles as well as of the adjacent ligaments and articular structures in between. After reconstructing the auditory ossicles from histological images based on the microgrinding technique, similar volumes of the same ossicles can be estimated (i.e.,* malleus* about 11800 *μ*m^3^,* incus* about 14910 *μ*m^3^, and* stapes* about 1660 *μ*m^3^).

Compared with the not calcified parts in the *μ*CT dataset the summarized volume of the cartilaginous and soft tissue inside the histologically reconstructed* malleus* (circa 150 *μ*m^3^) and inside the* incus* (circa 310 *μ*m^3^) reveals little higher values as 1.27% and 2.1%, respectively. This is probably due to the higher resolution and clear border lines of the histological structures. In this way very fine and small structures like collagen fibers, chondrocytes, and small vessels can be differentiated three-dimensionally (Figures 3(c) and 3(d)). The 3D model of the stained planes allows to analyze different orientated histological structures morphometrically; that is, in the* malleus* the vessels forming a fine, dense network in sagittal direction have diameter in head and neck in a range of 14 *μ*m to 83 *μ*m, whereas in the* manubrium* in a range of 14 *μ*m to 55 *μ*m (Figure 3(a)). Inside the* incus* a wide, branched out vessel network runs in transversal direction (Figure 3(b)), whereas in the long* crus* these blood vessels have a diameter in a range between 31 *μ*m and 35 *μ*m; inside the body their diameter varies from 14 *μ*m to 59 *μ*m.

In a study from Rau et al. [17], the geometric accuracy of the grinding method was compared with CT and micro-CT. Therefore, the intersectional distances of the ossicles were 100 *μ*m versus 35 *μ*m in the present study, which allows a 3D analysis of the histological properties of the fine structures in and around the ossicles in higher resolution. They used colored resin, which seemed beneficial for the fast determination of the borders of the evaluated structures, but for our purposes, for example, describing the run of the very little vessels inside the ossicles, transparent resin was needed. In the present work, vessels, cartilages, and ligament fibers could be segmented and modelled. Furthermore, Fuchsine was used for staining which marked only the nuclei of the cells. In the present study not only the whole cells but also the extracellular matrices could be evaluated after staining with the modified method according to Mann and Dominici.

Anson and Winch [7] are the only work group who reproduced the complex 3D structure of the intraosseous blood vessel system. They traced the images of photomicroscopy sections on pieces of cardboard with an enlargement factor (60x) and cut the outlines to stack together. The problem of the emerging asymmetries during the magnification process and furthermore the free hand cutting of the outlines gave a very high error index to the generated 3D model. Additionally, handmade pictures of the developed model were drawn for illustration.

The 3D model of the intraosseous blood vessel system of* malleus* and* incus* discussed in our study, however, was obtained by loading histological images after a very complex adjustment process in a CAD program and generates a highly accurate model of the found structures of the histological examination with a very low error index. Also, in using untainted, not decalcified bone we were able to rebuild the network of unshrunk blood vessel inside the auditory ossicles and could distinguish cartilage areas and blood vessels. Furthermore, measuring volume and the small diameter of the blood vessels becomes possible in our model. Anson and Winch [7] show either densely packed blood vessels or numerous centrally placed “core channel” of the blood vessels. These channel systems with large diameter cannot be seen in our histological 3D model, but inside the* incus* centrally placed cartilage areas are clearly separated (Figure 3). By evaluating the histological images of the microgrinding method, differences in the tissue composition of the auditory ossicles can not only be represented graphically, but also quantified.

## 4. Conclusion

Our study shows that the microgrinding method is necessary to get all anatomical characteristics of the human auditory ossicles. By comparing the histological images of the stained plane with a parallel look to the deeper zones of the specimen by using uncolored epoxy resin, the 3D reconstruction is possible in a very high resolution. For designing a 3D model of the middle ear structures, the distance between the planes has to measure about 34 *μ*m in order to allow the alignment of all fine structures inside the middle ear.

Whereas the time consuming microgrinding method and the high amount of workload in the adjustment process within the CAD program are necessary, the benefit becomes visible when compared with the results extracted from the *μ*CT analysis. The *μ*CT model shows only the calcified bone.

In contrast, the 3D model of the microgrinding images differentiates the histoanatomical structures and can rebuild, for example, the branched blood vessel system and the cartilage areas as well the course of collagen fibers. In order to substantiate these findings and for analyzing variations in anatomy, a second study with more specimens has to be undertaken.

An anatomically correct 3D model of the ossicular chain inside the intact middle ear is essential for the construction of physically optimized middle ear prostheses and for advanced middle ear surgery. In addition, it can be also used for computer based mechanical simulation of the sound transmission system.
